# High efficiency, high color purity red micro-light-emitting diodes

**DOI:** 10.1038/s41377-026-02227-3

**Published:** 2026-02-28

**Authors:** Yuanpeng Wu, Yixin Xiao, Maddaka Reddeppa, Yakshita Malhotra, Yifu Guo, Jianyang Xiao, Jiangnan Liu, Danhao Wang, Kai Sun, Zetian Mi

**Affiliations:** 1 Department of Electrical Engineering and Computer Science, University of Michigan, Ann Arbor, MI 48109 USA; 2 Department of Materials Science and Engineering, University of Michigan, Ann Arbor, MI 48109 USA

**Keywords:** Inorganic LEDs, Photonic crystals

## Abstract

InGaN-based micro-light-emitting diodes (micro-LEDs) are emerging to revolutionize the display and lighting technologies, particularly for their excellent robustness, high brightness, high efficiency and small pixel size. Despite the success of blue LEDs, long-wavelength emission, particularly the red emission, has been a challenge for InGaN-based micro-LEDs. Overcoming the low quantum efficiency, color instability, and broad emission in the red wavelength regime are among the most urgent and critical problems that inhibit the commercial implementation of micro-LED technology. In this work, we utilize a nanowire photonic crystal (PhC) structure to reform the radiation behavior of red-emitting InGaN micro-LEDs. Through detailed optimization on the PhC design and device fabrication, we demonstrate red-emitting micro-LEDs with a peak wavelength at 617 nm and a full-width-at-half-maximum (FWHM) of 5 nm, which is about one order of magnitude narrower than previous reported values and is paramount for achieving high color purity. The chromaticity property is highly stable with varying injection currents due to the coupling of emission to photonic band edge mode. A high external quantum efficiency of over 10% was measured from micro-LEDs with a size of 1 µm^2^. This work provides a vital strategy for high-performance red-emitting micro-LEDs and a potential pathway for full-color micro-LED technology by using all III-nitride semiconductors.

## Introduction

Micro-light-emitting-diodes (micro-LEDs) have attracted extensive research interests due to their emerging applications, including displays, virtual/augmented reality and visible-light communication^1–8^. The inherent features of InGaN, including excellent chemical robustness, wide bandgap tunability and relatively low surface recombination velocity, are the potential advantages for realizing blue, green and red micro-LEDs in one material system, which is highly desirable for monolithic integration and large-scale manufacturing of micro-LED displays^9–13^. Remarkable progress has been achieved in alleviating the efficiency deterioration during the scaling down of micro-LEDs by using techniques such as low-damage etching and surface passivation, particularly in the blue and green-emitting micro-LEDs^14–19^. Recently, while the external quantum efficiency (EQE) of blue and green InGaN micro-LED has increased significantly, the EQE of micrometer-scale red InGaN micro-LED is well below 1%, and few groups reported several percent EQE values, which can be mainly attributed to the high densities of defects and dislocations in high indium composition InGaN quantum wells (QW)^15,20–25^.

In addition to the EQE, the color purity is another major parameter to be optimized for displays yet received sufficient attention among the investigations conducted in InGaN micro-LEDs^1,26–28^. Micro-LEDs with high color purity hold great potential for transforming traditional displays due to their ability to achieve high contrast ratio, a wide color gamut, and excellent color saturation^26,29^. In general, high-color-rendering capabilities demand light sources with a full-width-at-half-maximum (FWHM) of <10 nm for all three primary colors as well as high color stability under various injection currents^29,30^. However, compositional segregation and a large built-in electric field in high indium composition InGaN QW causes significant blue-shift in emission color with injection current and broad emission with a typical FWHM of 40–90 nm^15,31–33^, preventing the realization of high-quality displays with appealing color rendering.

To overcome these challenges, nanowire-based architecture and photonic crystal (PhC) designs have been explored as promising strategies for enhancing LED performance^21,34–36^. Nanowire structures inherently provide advantages such as reduced etching-induced damage and enhanced strain relaxation, resulting in higher quantum efficiency and improved reliability^37,38^. Moreover, selective-area epitaxy enables the deterministic arrangement of nanowires into an ordered PhC structure, offering an additional degree of freedom to control light–matter interaction. PhCs, in particular, have been shown to tailor the spontaneous emission dynamics of semiconductors by coupling emission into guided or band edge modes, thereby improving both emission directionality and extraction efficiency^34,39^. Wierer et al., demonstrated that PhC architectures could yield extraction efficiencies as high as 73%^34^, while Sheen et al., reported that nanowire-based PhC design, along with effective sidewall passivation could dramatically enhance the EQE of InGaN emitters^21^. These studies highlight the unique potential of nanowire PhCs to improve the performance of InGaN micro-LEDs.

In this work, we utilize a nanowire PhC structure to significantly improve the performance of InGaN red-emitting micro-LEDs. The spontaneous emission characteristics of the micro-LED are controlled by coupling the emission of InGaN/GaN active region to the photonic band edge mode of the PhC. Pure red emission with narrow linewidth and negligible variation in emission color was obtained over a large range of injection currents. Through detailed optimization of the PhC design and device fabrication, high EQE values were demonstrated from a micrometer-scale InGaN LED.

## Results

**Fig. 1:**
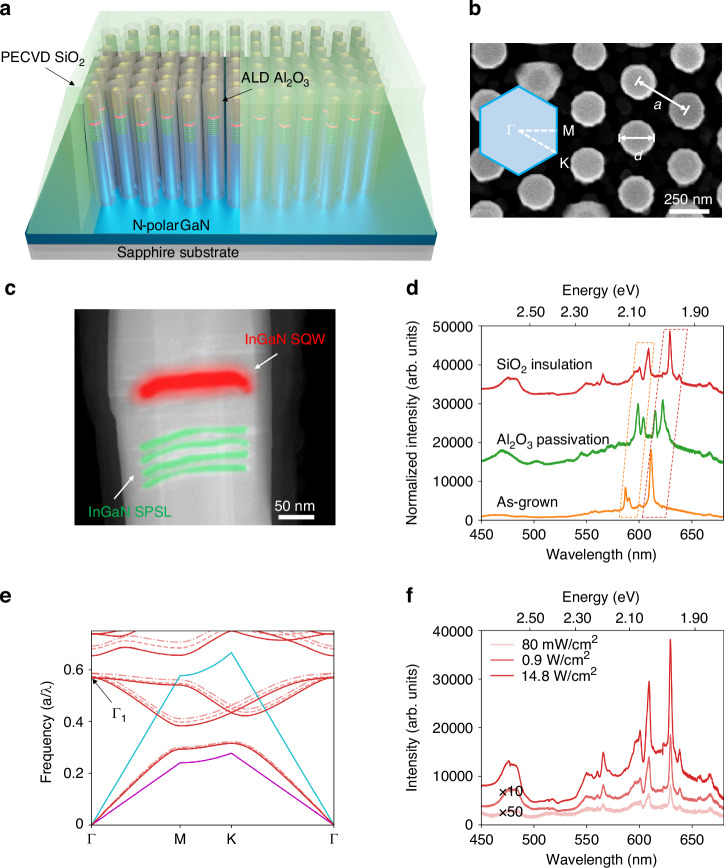
Structural and optical properties of nanowire InGaN/GaN heterostructures. **a** Schematic of the InGaN/GaN nanowire array arranged in a photonic crystal structure. The nanowires are enclosed by a thin layer of Al_2_O_3_ for surface passivation. The top of the nanowire array is covered by a thick SiO_2_ layer for insulation during the device fabrication. **b** Top view SEM image of the as-grown InGaN/GaN PhC structure with lattice constant *a* of 360 nm and diameter *d* of 220 nm. Inset: the corresponding schematic for the reciprocal lattice of the nanowire PhC structure. **c** Cross-sectional STEM image of the active region of the InGaN/GaN heterostructure. The red-emitting InGaN SQW and the InGaN/GaN SPSL are depicted by red and green false color, respectively. **d** Photoluminescence spectra measured from the as-grown sample (orange curve), after Al_2_O_3_ passivation (green curve) and SiO_2_ deposition (red curve). The evolution of the photonic crystal mode is indicated by the dashed rectangles. **e** Bandstructure of the nanowire photonic crystal structure without Al_2_O_3_ passivation (dash-dotted curves), with Al_2_O_3_ passivation (dashed curves) and with SiO_2_ insulation (solid curve). The light lines of GaN and air are shown as magenta and blue lines. **f** Power-dependent PL measurements of the structure with SiO_2_ and Al_2_O_3_ passivation over an excitation range of over two orders of magnitude

Figure 1a shows the schematic of the nanowire PhC structure with Al_2_O_3_ surface passivation deposited by atomic layer deposition (ALD) and SiO_2_ insulation layer deposited by plasma-enhanced chemical vapor deposition (PECVD). The epitaxy of the InGaN/GaN heterostructure was performed on a nitrogen-polar GaN on sapphire substrate with 10 nm patterned Ti mask. Prior to the growth of the red-emitting InGaN single quantum well (SQW), four periods of *n*-type doped InGaN/GaN short-period superlattices (SPSL) were grown to effectively release the strain and enhance the indium incorporation in the subsequent InGaN SQW growth. A top-view scanning electron microscope (SEM) image of the as-grown nanowire array with the lattice constant *a* and diameter *d* labeled is shown in Fig. 1b. Figure 1c illustrates the cross-sectional scanning transmission electron microscope (STEM) image of the active region of the InGaN/GaN heterostructure, wherein the red false-colored region depicts the InGaN SQW and the green false-colored region depicts the SPSL. The indium map of the InGaN active region is shown in Fig. S1 of the Supporting Information, and the estimated indium composition on *c*-plane and semipolar-plane are ~36% and 40%, respectively. The selective area growth of the bottom-up nanowire array and the related substrate patterning procedure can be found in our previous reports^12,17,25,40,41^ and in Methods. Figure 1d compares the normalized photoluminescence spectra of a nanowire PhC with *a* of 360 nm and *d* of 220 nm under different passivation conditions. Multiple sharp emission lines were observed from the as-grown nanowire PhC array. The dominant mode is at 612 nm with FWHM of ~3 nm, which corresponds to the photonic band edge mode (Γ_1_) as indicated in the calculated photonic bandstructure in Fig. 1e. The peaks at 587 nm and 591 nm can be attributed to different photonic band edge modes.

After the surface passivation of 10 nm Al_2_O_3_, two peaks around 616 nm and 623 nm were observed while the shorter wavelength peaks shifted to 599 nm and 604 nm, respectively, along with the alternations in the relative peak intensities. The solid red lines in Fig. 1e are the photonic band structure of Al_2_O_3_ passivated PhC structure, wherein the normalized frequency at Γ_1_ point shifts down by 0.013, which agrees with the red shift of ~10 nm in the peak positions. ALD-based surface passivation has been widely used for reducing the leakage current through the etching-induced damages on the sidewalls in micro-LEDs^42–44^. Other passivation techniques, such as the sol-gel method, were also reported^21^. Al_2_O_3_ passivation with thicknesses of 5 nm, 20 nm and 60 nm has been adopted in the samples with nominally the same growth conditions, and no pronounced optical resonance was observed using 20 nm and 60 nm Al_2_O_3_ passivation as shown in Fig. S2 of the Supporting Information. The thickness of the sidewall passivation layer greatly affects the geometry and the distribution of refractive index of the PhC structure, wherein a thicker passivation layer reduces the refractive index contrast between InGaN/GaN nanowire and the gaps as well as the PhC effect. Additional discussions about the effect of Al_2_O_3_ passivation on the resonant modes can be found in Section III of the Supporting Information. The red curve in Fig. 1d is the PL spectrum of the nanowire PhC measured after an additional 300 nm SiO_2_ deposition, wherein the SiO_2_ functions as the insulation layer during the metallization of electrodes. The dominant peak further shifts to 629 nm while the Γ_1_ point shifts down by 0.004, as shown by the solid red line in Fig. 1e. Such a shift can be attributed to the variation of the refractive index distribution, wherein the presence of the SiO_2_ increases the average background refractive index. The evolution of the peaks with different passivation or insulation conditions is indicated by the dashed boxes in Fig. 1c. Red shifts with similar trends have been consistently observed from patterns with different designs.

Figure 1f shows the power-dependent PL measurements performed on the nanowire PhC structure after Al_2_O_3_ and SiO_2_ deposition, wherein the emission peak is invariant with the excitation power varied over two orders of magnitude, which is in direct contrast to the significant blue-shift of 20–50 nm observed in conventional red InGaN LEDs due to the screening of the quantum-confined Stark effect^1,45,46^. The excited carriers within the InGaN SQW generate spontaneous emission coupled to the photonic band edge mode of the PhC structure, wherein the emission wavelength is insensitive to the carrier density, and a negligible shift in peak wavelength is obtained.

**Fig. 2:**
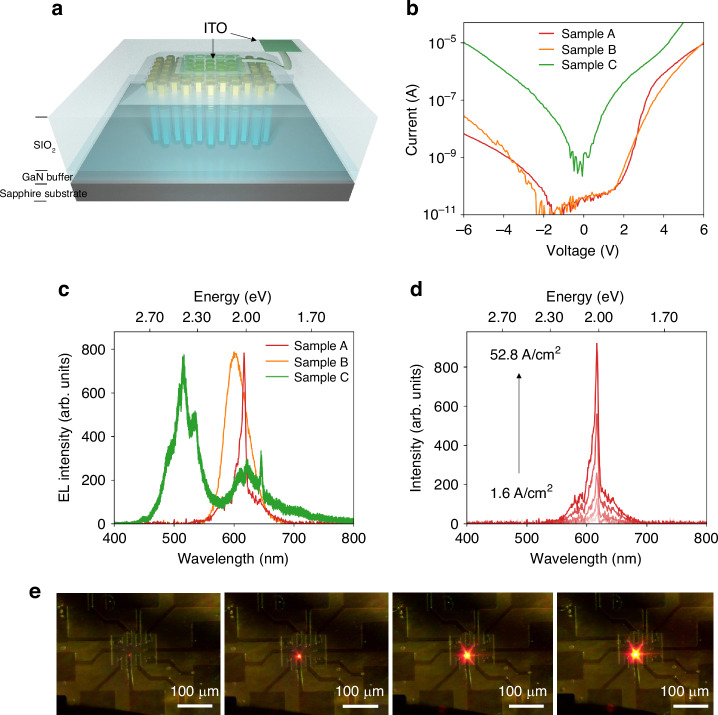
Performance characteristics of nanowire PhC micro-LED under different surface passivation conditions. **a** Schematic of the InGaN/GaN micro-LED device structure. **b** Current-voltage characteristics of the devices from Sample A, B and C. **c** Normalized EL spectra of the devices from Sample A, B and C. **d** Current-dependent EL spectra for the micro-LED from Sample A. **e** The optical microscope image of the red-emitting micro-LED under injection currents of 0.36 A/cm^2^, 1.85 A/cm^2^, 5.9 A/cm^2^ and 7.8 A/cm^2^

Following the deposition of the SiO_2_ layer, photolithography followed by a fluorine-based RIE etching process was performed to define the current injection windows with sizes ranging from sub-micrometre scale to 8 µm × 8 µm. The metallization includes ITO for the *p*-contact and Ti/Au for the *n*-contact. Figure 2a is the schematic of the as-fabricated nanowire PhC micro-LED. Three samples of InGaN/GaN nanowire PhC were grown under nominally identical conditions and fabricated using the same processing except with Al_2_O_3_ thicknesses of 10 nm (Sample A) and 60 nm (Sample B) and without Al_2_O_3_ passivation (Sample C). A SEM image of a 1 µm^2^ current injection window is shown in Fig. S4 in the Supporting Information. Detailed fabrication parameters can be found in Methods. Figure 2b compares the current-voltage (*I–V*) characteristics of micro-LEDs with a size of 1 µm^2^ from the three samples, wherein the measured device in Sample A has *a* of 320 nm and *d* of 240 nm, and the devices in Sample B and C have *a* of 360 nm and *d* of 220 nm. Current densities of Samples A and B are similar and are one to two orders of magnitude lower than that of Sample C. Previous reports have adopted ALD thin films with thicknesses ranging from 7 to 30 nm for sidewall passivation in micro-LEDs^43,44^. The sidewall passivation by Al_2_O_3_ and the corresponding electron dispersive spectroscopy analysis can be found in Section V of the Supporting Information. Meanwhile, the rectification ratio of Samples A and B at ±6 V is two orders of magnitude higher than that without passivation (Sample C). The reduction in current density at both forward and reverse bias with ALD thin-film passivation had been observed before and was attributed to a reduction in sidewall surface state and fewer trap states for charge carriers^16,18^. Figure 2c shows the normalized electroluminescence (EL) spectra of the three samples, wherein the emissions from the InGaN/GaN short superlattice are absent, and only the emissions from the red-emitting InGaN SQW are present for Sample A and Sample B. The FWHM of the EL of Sample B is ~50 nm, which is comparable to the 45-80 nm linewidths reported from other InGaN red-emitting LEDs^15,31,32^. The EL spectrum of Sample A contrasts that of Sample B and exhibits a dominant emission peak at 617 nm. The sharp peak has a FWHM of ~5 nm, which is about one order of magnitude narrower than that of the previous reports. A red-emitting sharp line at 645 nm is observed from Sample C as well. However, a broad emission with a peak at 512 nm was observed from Sample C, which can be attributed in part to the emissions from the InGaN/GaN short superlattice. Considering that the high current density and poor rectification ratio measured from Sample C, the emission from the superlattice can be attributed to the leakage current from the sidewall.

Current dependent EL measurements were performed on Sample A and it can be seen from Fig. 2d that the peak wavelength is nearly invariant when the injection current increases by over one order of magnitude, The excellent color stability was further confirmed by the optical images of a 5 µm × 5 µm red-emitting micro-LED device under injection current densities from 0.36 A/cm^2^ to 7.8 A/cm^2^ as shown in Fig. 2e, wherein no variations in emission color was observed. Moreover, red rays radiating from the micro-LED were seen to propagate along the six equivalent Γ-M directions in the surrounding PhC region. The observation of propagating light is possibly due to some surface roughness between the SiO_2_ insulation layer and the nanowire top. Such a radiation pattern is absent in Sample B, where much thicker Al_2_O_3_ passivation is used.

**Fig. 3:**
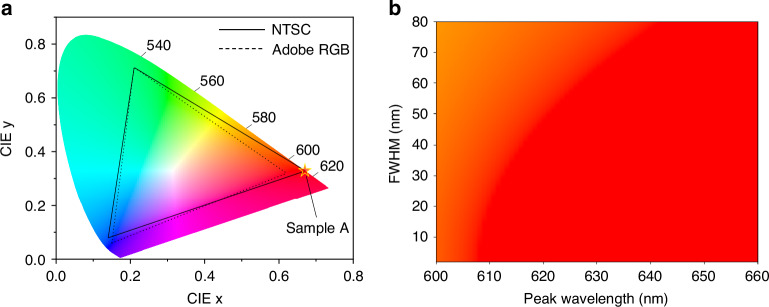
Chromatic properties of the red-emitting micro-LED and the effect of the spectral linewidth on the chromaticity. **a** Color point created from the red micro-LEDs of Sample A plotted on the 1931 CIE chromaticity diagram. The color gamut of NTSC standard and Adobe RGB is also indicated on the diagram. **b** Chromaticity diagram of emission spectrum with Gaussian distribution at various peak FWHM and wavelength in the red wavelength regime

The chromaticity coordinate of the red-emitting micro-LED in Sample A is (0.67, 0.33), which is marked as a red star in the International Commission on Illumination (CIE) 1931 chromaticity diagram as shown in Fig. 3a. This coordinate coincides with that of the primary red color in the widely used National Television Standards Committee (NTSC) standard and is beyond the color gamut area of the Adobe RGB. In previously reported InGaN red-emitting micro-LEDs, while excellent color purity can be achieved at low current densities of a few A/cm^2^, the chromaticity coordinates deviate from the red region, and the color gamut coverage reduces due to the significant blueshift with injection current^26,28^. In the presented micro-LED based on InGaN nanowire PhC, the chromaticity coordinate remains almost constant when the micro-LEDs were driven at different current densities due to the negligible shift in peak wavelength. The effect of the spectral linewidth on the chromaticity of red-emitting micro-LEDs is illustrated by plotting the chromaticity of the luminescence spectra with a Gaussian distribution in the wavelength range of 600–660 nm and FWHM range of 2–80 nm. As shown in Fig. 3b, the FWHM plays a significant role in deviating the color from red to amber when the peak wavelength is below 630 nm. Namely, emission peak wavelengths of over 630 nm are desirable for achieving pure red emission at a typically observed FWHM of ~60 nm from InGaN micro-LEDs. However, such a long emission wavelength requires a high indium composition of over 50%, which could lead to high densities of defects and dislocations in the InGaN/GaN QWs and greatly deteriorate the EQE.

**Fig. 4:**
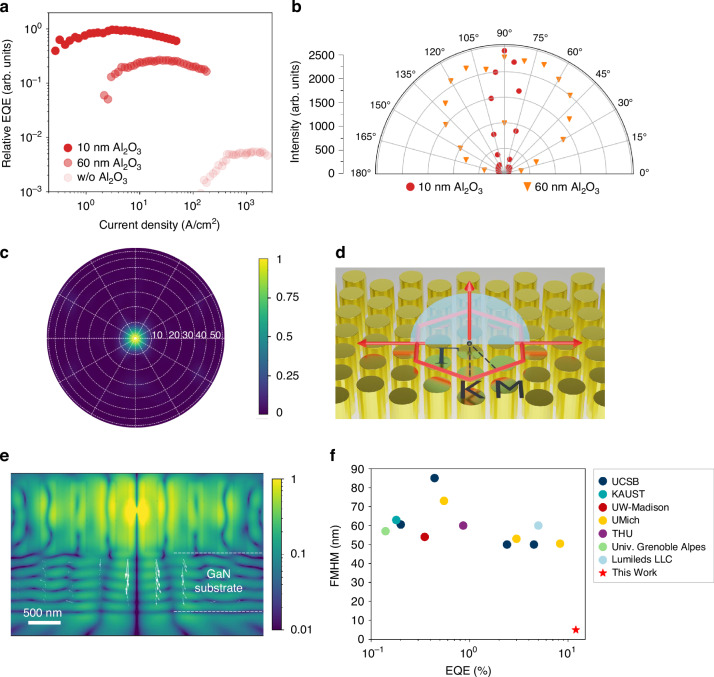
EQE, far-field emission distribution and electric field profile of the red-emitting micro-LEDs. **a** Measured relative EQE of devices with different surface passivation conditions. **b** Far-field angular distribution of EL intensity. **c** Calculated far-field radiation pattern of a PhC structure with *a* of 320 nm, *d* of 240 nm and Al_2_O_3_ passivation of 10 nm. **d** Schematic showing propagating directions of coupled waves along the Γ-M directions. **e** Calculated electric field distribution within the PhC structure. The white arrows are the Poynting vectors in the GaN substrate. **f** Benchmark of EQE value and linewidth value for some previously InGaN-based red micro-LEDs with device areas ≤ 400 µm^2^ ^12,15–17,24,48–55^

The devices in Samples A, B and C were further fabricated by depositing metal reflectors consisting of 150 nm Al/10 nm Ti/20 nm Au on top of the ITO for EQE measurements. As shown in Fig. 4a, a peak EQE of Sample A is about four times that of the device in Sample B and is more than two orders of magnitude larger than that of the device in Sample C. The measured EQE of Sample A peaks at a current density of 4 A/cm^2^, which is about two to three orders of magnitude lower than that of Sample C. A linear EQE plot is shown in Fig. S6 in the Supporting Information. Previous reports have shown that leakage current causes a low EQE and higher peak EQE current density, which is further confirmed by the lack of apparent droop in the EQE as observed from Sample C^47^. The obtained peak EQE from Sample A is ~12%, wherein the samples were placed on top of a large area silicon detector for optical power measurements. Such an EQE measurement strategy has been adopted in our previous reports^12,17,25,40^. The schematic of the EQE measurement setup and the calibration procedure can be found in Fig. S7 of the Supporting Information. Angular-resolved EL measurements were performed to investigate the far-field emission distribution of the micro-LEDs in Sample A and Sample B, and the results are shown in Fig. 4b. The EL emission from the device in Sample A is mainly distributed along the direction that is perpendicular to the device surface, within a divergence angle of less than 20°. In contrast, the EL collected from Sample B follows a distribution that is close to a Lambertian distribution. Optical simulations based on three-dimensional finite-difference time-domain (FDTD) were performed on a nanowire PhC structure with the same design as Sample A. Detailed simulation parameters can be found in Section VIII of the Supporting Information. The calculated far-field radiation pattern of the photonic band edge mode features a divergence angle of <10°, as shown in Fig. 4c. The larger divergence angle observed experimentally than the theoretical simulation can be attributed to the scattering of light into unintended directions by the nanowires with imperfections in the shape, as shown in Fig. S9 of the Supporting Information. The vertical emission mechanism is illustrated in Fig. 4d, wherein the lightwaves that propagate in the forward and backward directions along the Γ-M directions couple together and their wave vectors satisfy the first-order Bragg condition, resulting in the redistribution of photonic energy in the vertical direction. Figure 4e shows the electric field profile of the photonic band edge mode, wherein the calculated Poynting vectors in the GaN substrate are predominantly downward-pointing. In comparison, for the same PhC structure with 60 nm Al_2_O_3_ passivation, the Poynting vectors within the GaN substrate have significant in-plane components (Fig. S9), indicating the formation of a guiding mode, which eventually dissipates and limits the light extraction efficiency. Using the PhC structure to improve the efficiency of large-scale blue-emitting LEDs has been demonstrated by Wierer et al., before, despite no narrowing of the linewidth being observed^34^. Figure 4f marks the EQE measured from this work compared with the reported red-emitting micro-LEDs with device area ≤400 µm^2^. To our best knowledge, this study shows the highest EQE values and smallest linewidth for red-emitting InGaN micro-LEDs.

The demonstrated nanowire PhC red micro-LEDs open up important opportunities for practical applications. First, the near-diffraction-limited emission area and ability to form large, ordered arrays make them highly promising for ultra-high-resolution micro-displays in augmented/virtual/mixed reality. The narrow linewidth emission and directionality of the photonic band-edge modes are advantageous for visible-light communication, wherein the reduced spectral crosstalk and high modulation bandwidth expected from nanoscale emitters could support high-speed, low-latency optical data links in next-generation wireless systems.

## Discussion

In conclusion, state-of-the-art red micro-LEDs with high efficiency and color purity have been demonstrated, which is pivotal for the future development of micro-LED technology. We show that through coupling the spontaneous emission of InGaN/GaN heterostructure to the photonic band edge mode of a nanowire PhC structure, pure red emission with narrow emission linewidths of 5 nm can be achieved, and no variations in emission color was observed over a wide range of injection currents. The chromaticity coordinate of the red micro-LED reaches the primary red color in the NTSC standard, which indicates excellent color-rendering potential over a large color gamut area. Directional emissions primarily along the vertical direction of the nanowire PhC structure were measured, which effectively improved the light extraction efficiency and a high EQE of 12% was obtained from micrometer scale red micro-LEDs. The excellent optical performance of the InGaN red micro-LEDs paves the way for monolithic integration of GaN-based RGB-pixelated micro-LEDs with CMOS-based driver circuitry.

## Materials and methods

### Epitaxial growth

The growth was performed on N-polar GaN on a sapphire substrate. Prior to growth, the substrate was first coated with 10 nm Ti film, which was subsequently patterned by e-beam lithography and an etching process to create a triangular lattice of GaN holes. After standard solvent cleaning, the patterned wafer was loaded into the MBE system for outgassing and subsequent nanowire micro-LED growth. The growth starts with the *n*-GaN segment at 680 °C, Gallium beam equivalent pressure (BEP) of ~3 × 10^−7 ^Torr, and nitrogen flow of 0.5 sccm. Prior to the growth of the red-emitting InGaN active region, four periods of Si-doped InGaN/GaN SPSL were grown to effectively release the strain and enhance the indium incorporation in the subsequent InGaN SQW growth. The active region was grown at a substrate temperature of 570 °C, gallium BEP of ~6 × 10^−8^ Torr, and indium BEP of ~1 × 10^−7 ^Torr. The nitrogen flow rate was 0.7 sccm for enhanced indium incorporation during active region growth. The *p*-GaN was grown at 680 °C for 60 mins. Mg BEP of 2 × 10^−8^ Torr was used.

### Fabrication

The fabrication starts with ALD of Al_2_O_3_ for sidewall passivation, followed by a fluorine-based reactive ion etching (RIE) process to reveal the top of the *p*-GaN. Then, 300 nm SiO_2_ was deposited by PECVD as the insulation layer, followed by lithography and another RIE etching to open the current injection window for each submicrometer/micrometer scale LED. The *p*-metal contact consists of 180 nm ITO deposited via a sputtering process, which provides excellent coverage on the sidewall of the SiO_2_ insulation layer. Chlorine-based RIE process on *n*-GaN was performed, followed by deposition of 20 nm Ti/80 nm Au for *n*-metal contact. Annealing in N_2_ ambient was performed at 500 °C for 1 min. Samples B and C were processed using similar processes except that Al_2_O_3_ of 60 nm was deposited on Sample B, and no Al_2_O_3_ passivation was deposited on Sample C. The top metal reflector consisting of 150 nm Al/10 nm Ti/20 nm Au was deposited by e-beam evaporator.

### Characterization

*I–V* characteristics of the as-fabricated micro-LEDs were measured using a Keithley 2400 voltage source meter. PL characterizations were performed by 405 nm laser excitation. The emissions from the nanowire array were collected by an optical fiber placed on top of the sample to collect the emission in a direction perpendicular to the PhC surface. The EL spectra were also collected by an optical fiber. The emissions were spectrally resolved by high-resolution spectrometers equipped with liquid nitrogen/thermally electrically cooled CCD cameras. The angle-dependent EL spectra were collected using a fiber mounted on a rotation stage. STEM characterizations of the samples were performed using a FEI Spectra Ultra Scanning Transmission Electron Microscope with double Cs-correctors and EDS mapping operated at 300 keV.

### Simulations

The photonic bandstructure is calculated for transverse magnetic polarization using the 3D FDTD method. PhC dimensions were selected by photonic-band calculations targeting a $\Gamma$-point resonance. The lattice constant $a$ sets the spectral position (${\lambda }_{\Gamma }\propto a$); the nanowire diameter $d$ adjusts the fill factor and effective index, providing further tuning of the emission wavelength. The electric field distribution profiles are calculated using the 3D FDTD method. Detailed simulation parameters can be found in Section VI of the Supporting Information.

## Supplementary information

Supplementary Information for High-Efficiency, High Color Purity Red Micro-Light-Emitting Diodes

## Data Availability

The data that support the findings of this study are available from the corresponding authors upon request.
