# Supplementary material for: High efficiency, high color purity red micro-light-emitting diodes
Source: Light Sci Appl. 2026 Feb 28;15:133. doi: 10.1038/s41377-026-02227-3 (PMC12949992; doi:10.1038/s41377-026-02227-3)
Supplement: Supplementary file 1 — Supplementary Information for High-Efficiency, High Color Purity Red Micro-Light-Emitting Diodes [file 41377_2026_2227_MOESM1_ESM.pdf]

Supplementary Information for
High Efficiency, High Color Purity Red Micro-Light-Emitting Diodes

Yuanpeng Wu[1,*], Yixin Xiao[1], Maddaka Reddeppa[1], Yakshita Malhotra[1], Yifu Guo[1], Jianyang Xiao[1], Jiangnan Liu[1], Danhao Wang[1], Kai Sun[2], and Zetian Mi[1,*]

[1)]Department of Electrical Engineering and Computer Science, University of Michigan, Ann Arbor, MI 48109, USA

[2)]Department of Materials Science and Engineering, University of Michigan, Ann Arbor, MI 48109, USA

[*)]Corresponding author: ypwu@umich.edu; ztmi@umich.edu

Section I. Indium composition characterization

The indium map of the InGaN active region, shown in Figure S1, shows an estimated indium composition of around 36% and 40% from c-plane (Point 1) and semipolar-plane (Point 2), respectively.

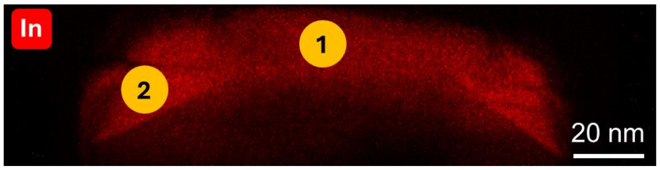

Figure S1. An EDS map for indium in the active region consisting of an InGaN single quantum well.

Section II. Effect of surface passivation on photoluminescence properties

Figure S2 shows the PL spectra comparison for 4 nanowire samples with Al_2O_3 passivation layer thicknesses of 5 nm, 10 nm, 20 nm and 60 nm, respectively. The blue curves are measured from the samples before Al_2O_3 passivation and the red curves are measured from the samples after Al_2O_3 passivation. It can be seen that with 5 nm passivation, the dominant peak of 607.3 nm shifted to 613 nm while with 10 nm passivation, the dominant peak of 612 nm shifts to 623 nm. For the samples with passivation thicknesses of 20 nm and 60 nm, the dominant peak disappears or weakens significantly after the passivation.

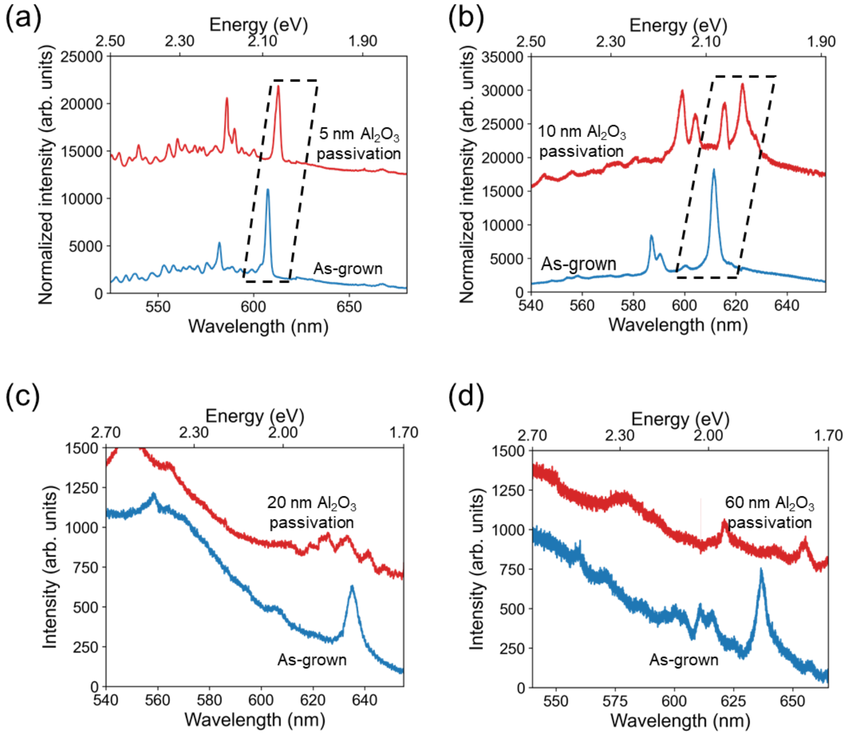

Figure S2. Photoluminescence spectra measured from nanowire photonic crystal arrays before passivation and after (a) 5 nm, (b) 10 nm, (c) 20 nm, and (d) 60 nm Al_2O_3 passivation.

The samples used for comparing the PL properties before and after Al_2O_3 passivation were grown using nominally the same conditions as the sample used in Figure 1d. However, the variations in the growth temperature and other factors result in different PL properties. For example, the sample used for Figure S2c has much stronger emissions from the short period superlattice than that from the red-emitting InGaN single quantum well. The purpose of Figure S2 is to show that Al_2O_3 passivation will cause the red-shift/weakening/disappearance of the red-emitting resonant mode. Therefore, the variation in the PL of the as-grown sample does not change the conclusion.

Section III. Calculation of the passivation effect on the optical properties of the PhC structure

The calculated photonic bandstructure shows a monotonic downward-shift of the Γ_1 band-edge with increasing Al_2O_3 thickness (Figure S3a), in agreement with the redshift in emission peak wavelength observed from PL measurement.

The disappearance of resonant peaks can be attributed to that, with increasing Al_2O_3 thickness, a significant amount of the light starts having a lateral component in the wavevector which facilitate the coupling of light emission into guiding mode within the GaN substrate, as shown in the 3D FDTD simulation in Figure S3b.

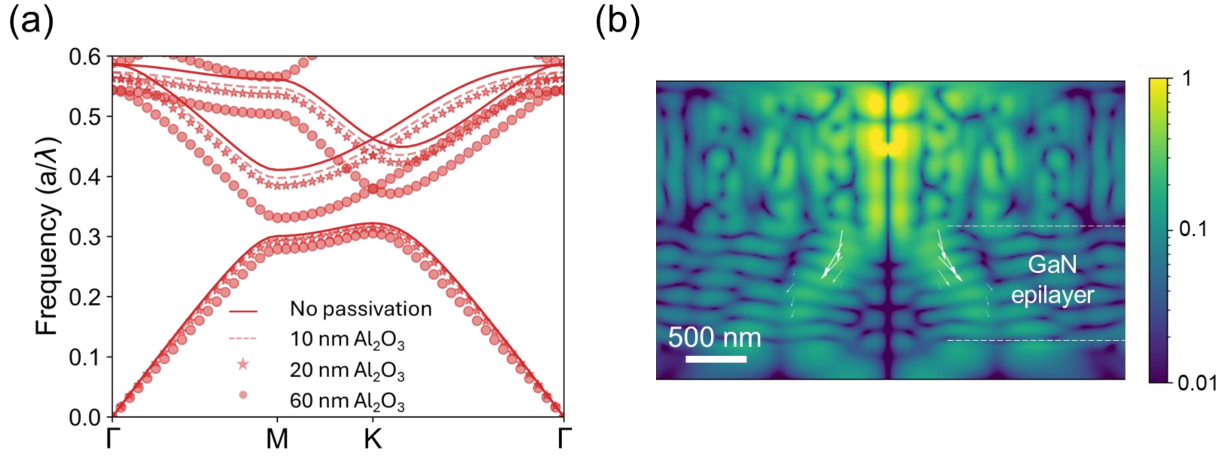

Figure S3. Calculated photonic bandstructure of InGaN/GaN nanowire array with Al_2O_3 passivation of different thicknesses. (b) Calculated electric filed distribution within the PhC structure (*a* of 360 nm, *d* of 220 nm and Al_2O_3 thickness of 60 nm). The white arrows are the Poynting vectors in the GaN substrate.

Section IV. Characterization of the current injection window of the red micro-LED in Sample A

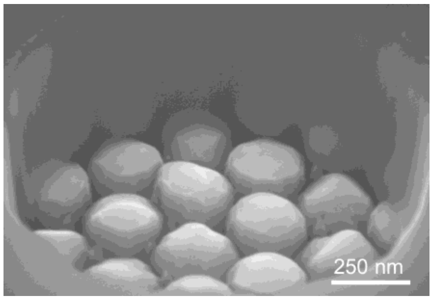

Figure S4. SEM image of a micro-LED in Sample A with a current injection window size of 1 μm^2.

Section V. Characterization of Al_2O_3 shell

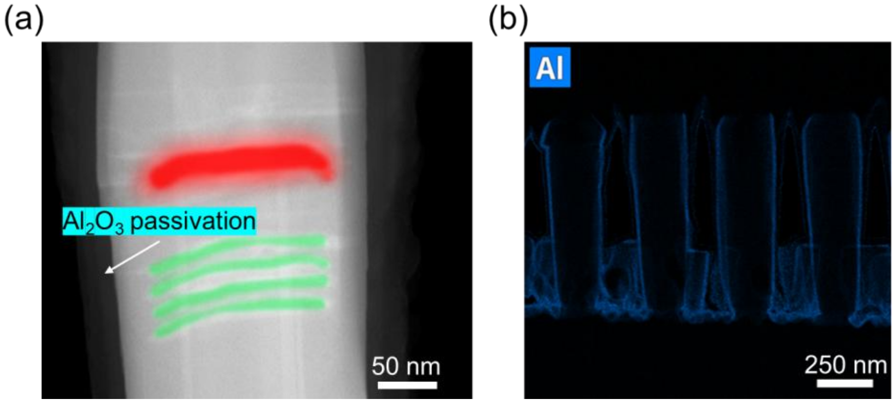

Figure S5. (a) Cross-sectional dark field transmission electron microscope image of the InGaN/GaN nanowire with Al_2O_3 passivation. The Al_2O_3 layer on the sidewall of the nanowire is indicated by the white arrow. (b) Electron dispersive spectroscopy elemental analysis showing the Al signal measured from the sidewall of the nanowires.

Section VI. Relative EQE plot in linear scale

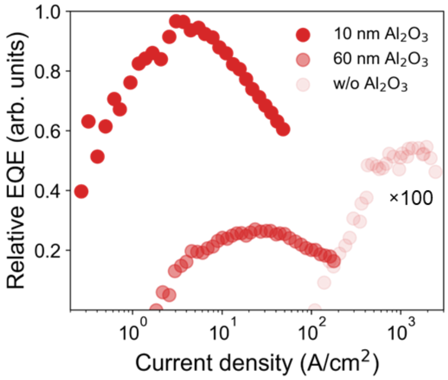

Figure S6. Measured relative EQE of devices with different surface passivation conditions.

Section VII. Setup for external quantum efficiency measurement

The external quantum efficiency measurements were performed by placing Samples A, B and C on a power detector without any packaging. Metal reflectors consisting of 150 nm Al/10 nm Ti/20 nm Au were deposited on top of the ITO for better light collection.

The external quantum efficiency is obtained based on the measured optical power and the current of the red-emitting micro-LEDs through:

$$\eta = \frac{P/h\nu}{I/e}$$

The optical power measurements used a NIST calibrated Newport 918D-ST-UV power detector, with a minimum measurable power of 20 pW. To verify the accuracy of the power meter, a directional LED source with known output power at different operating currents was used to cross check the response of the power detector. This procedure allowed us to ensure the absolute optical power with an estimated uncertainty below 5%. The current of the red micro-LED was measured using a Keithley 2400 source meter with current resolution down to 10 pA, which ensures the accuracy of current measurements.

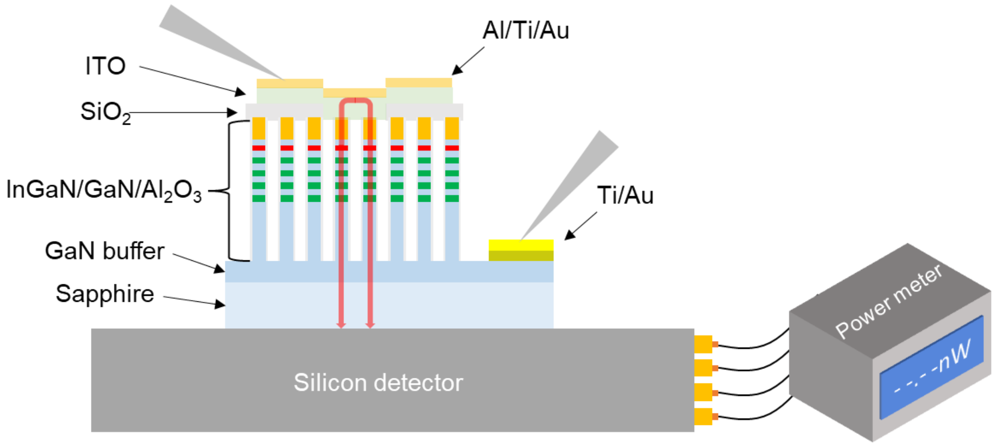

Figure S7. Schematic of measurement setup for the external quantum efficiency.

The external quantum efficiency measurements were performed by placing Samples A, B and C on a detector. Metal reflectors consisting of 150 nm Al/10 nm Ti/20 nm Au were deposited on top of the ITO for better light collection.

Section VIII. Finite-Difference Time-Domain (FDTD) simulations of nanowire PhC structures

We have performed FDTD simulation using photonic crystal structure with the same design as Sample A (*a* of 320 nm and *d* of 240 nm) and Sample B (*a* of 360 nm and *d* of 220 nm). The length of the p-GaN, InGaN/GaN active region, InGaN/GaN short period superlattice (SPSL) and n-GaN are 180 nm, 50 nm, 100 nm and 500 nm, respectively. The refractive index of 1.76, 2.38, 2.56 and 2.6 are used for the Al_2O_3, GaN, SPSL and active region, respectively [1, 2]. Below in Figure S8, we show that for the PhC structure with 60 nm Al_2O_3 shell, the calculated Poynting vector within the GaN substrate has a significant in-plane component, indicating the formation of guiding mode within the GaN substrate.

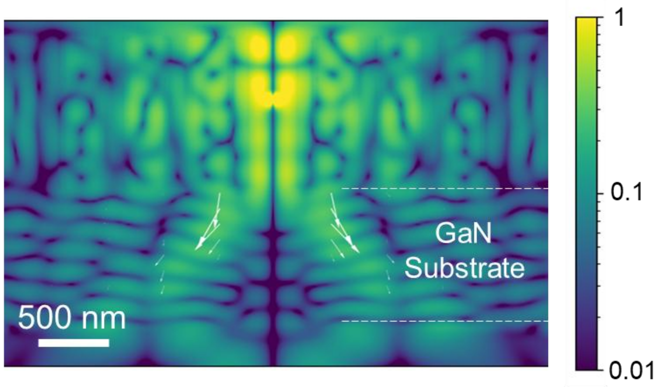

Figure S8. Calculated electric filed distribution within the PhC structure. The while arrows are the Poynting vectors in the GaN substrate.

Section IX. SEM characterization of the nanowire photonic crystal array

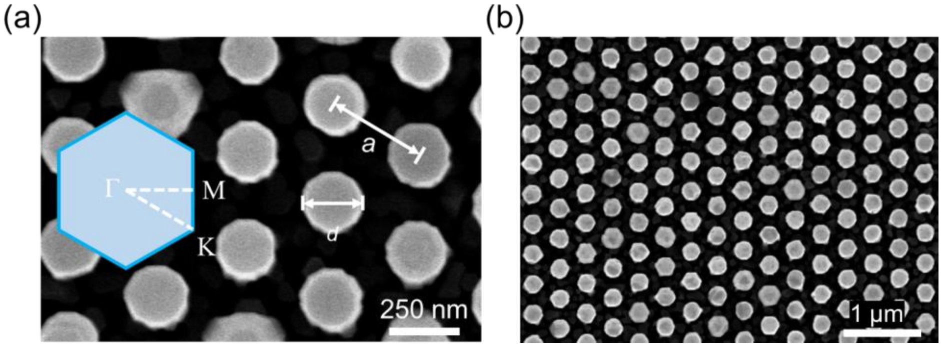

Figure S9. Top view SEM images of InGaN/GaN nanowires arranged into a photonic crystal structure.

References:

1. Ra YH, Rashid RT, Liu X, Sadaf SM, Mashooq K, & Mi Z, An electrically pumped surface-emitting semiconductor green laser. Sci Adv, 2020, 6(1):eaav7523.
2. Polyanskiy MN, Refractiveindex.info database of optical constants. Sci Data, 2024, 11(1):94.
